# The Design and Maintenance of Low-Orbit Navigation Constellation for Traffic Control in a Smart City

**DOI:** 10.3390/s22239478

**Published:** 2022-12-04

**Authors:** Yi Zheng, Baojun Lin, Rui Li, Yutong Liu

**Affiliations:** 1 University of Chinese Academy of Sciences, Beijing 100049, China; 2 Aerospace Information Research Institute, Chinese Academy of Sciences, Beijing 100094, China; 3 Innovation Academy for Microsatellites, Chinese Academy of Sciences, Shanghai 201210, China; 4 Shanghai Engineering Center for Microsatellites, Shanghai 201304, China; 5 School of Information Science and Technology, Shanghai Tech University, Shanghai 201210, China; 6 School of Electronic Information and Electrical Engineering, Shanghai Jiao Tong University, Shanghai 200240, China

**Keywords:** smart city, traffic control, LEO satellite, navigation constellation, atmospheric drag, station keeping

## Abstract

The traffic control issue in the smart city scenario gives rise to the higher requirements of Global Navigation Satellite System (GNSS) services, especially in terms of navigation accuracy, together with coverage continuity, and multiplicity. The dense urban environment leads to higher elevation angles for navigation in such areas, which requires a lower altitude of the constellation, as well as a larger number of satellites. In the existing literature, the design and maintenance of the Low Earth Orbit (LEO) navigation constellation that fulfills the requirements of the smart city are not provided. Hence, based on the requirements and constraints of the smart city scenario, this article studies the relation between orbital height, user elevation angle, and coverage. It designs the configuration of an LEO navigation constellation that not only achieves global sensing coverage, but also provides a continuous lane-level navigation service with multiple coverages for the key area. In addition, considering the atmospheric drag in low orbits and the constraint of satellite power and attitude control, a method is proposed by rotating solar panels to change the effective frontal area of the satellite to achieve relative configuration maintenance of the LEO constellation. The results show that the LEO navigation constellation has a 0 s revisit time in five chosen smart cities, and each city has more than four-times coverage every second; the Geographic Dilution of Precision (GDOP) values of five cities are smaller than 0.47. The average navigation accuracy of five cities is 2.01. With the conduction of the one-year station-keeping simulation, the phase deviation of two satellites is less than 0.6° and it gradually converges to 0.1°, where the semi-major axis deviation is less than 80 m. With our proposed method, the active station-keeping control is not needed in one year, and the fuel consumption can be reduced. Finally, the continuity of the navigation service can be assured.

## 1. Introduction

Major cities around the globe are becoming increasingly crowded with inhabitants and vehicles, leading to an increase in traffic. As one of the important smart city sensing applications, smart traffic control with congestion monitoring and accident detection capability highly relies on vehicle positioning and navigation services [1]. In the smart city scenario, to successfully monitor road conditions and relieve congestion, it is important for the public transport control center as well as the personal user to acquire lane-level road conditions. For public users, such information allows them to achieve smart bus route planning. Since the designated bus route cannot be changed at will, real-time lane-level navigation information allows them to choose the optimal lane to avoid congestion [2]. In addition, emergency information such as traffic accidents and rescues could be transmitted through the global satellite network with inter-satellite communications and efficient constellation configuration to ensure their availability, and lane-level road information is also critical for first-aid personnel to arrive on time [3]. For personal users, traffic-related applications in the smart city scenario such as intelligent driving, unmanned driving, and real-time parking space monitoring require higher navigation accuracy, as well as positioning precision. These applications not only help residents enjoy a better driving experience, but also aid in relieving congestion. According to the traffic control issue discussed above, navigation information, as one of the major sensing data required in the smart city, is facing an unprecedented challenge. Such data should possess real-time lane-level navigation accuracy, and it is necessary to ensure the continuous availability of all road conditions under complex terrain, such as the interference of high-rise buildings [4]. The above scenarios propose higher requirements for the accuracy, integrity, and real-time convergence time of navigation services. However, the four existing satellite navigation systems, Galileo, GPS, BDS, and GLONASS, cannot satisfy such high requirements [5]. First, the traditional turn-by-turn (TBT) navigation only reaches road level, and its positioning accuracy provided by the current GNSS basic navigation service is usually only about 10 m [6]; however, the traffic-related applications in the smart city scenario require higher navigation accuracy, as well as positioning precision. Second, the constellation of the four major GNSS consists of numerous high- and medium-orbit satellites; the long transmission distance leads to a weak GNSS signal, which is not sufficient to penetrate the physical barrier in some scenarios such as forests, metropolis, canyons, and other signal-shielded areas. In addition, GNSS signal power is so low that it can easily be interfered with and deceived, which creates certain hidden dangers [7]. Moreover, the Precise Point Positioning (PPP) service provided by the existing GNSS still relies on Medium Earth Orbit (MEO) and High Earth Orbit (HEO) navigation satellites, and its geometric configuration between satellites and ground users changes so slowly that it takes more than 10 min to achieve meter-level positioning accuracy. If the LEO satellite is used for PPP service, the convergence time could be reduced to seconds [8]. Therefore, to meet the requirements of satellite navigation in a smart city, we explore an efficient LEO navigation constellation construction to further provide the accuracy, integrity, and real-time services.

Since the existing medium- and high-orbit navigation systems cannot meet the needs, low-orbit constellation seems to be more effective as a satellite-based navigation platform. The lower orbit and lighter mass enable carrier rockets to launch efficiently and quickly in the way of one rocket with multiple satellites. In addition, due to a shorter signal transmission distance and less free space loss, it helps to improve the positioning effect in the signal-shielded environment and improve the anti-interference and anti-deception performance. In the case of how to convey the navigation signal by using the LEO constellation, Rabinowitz [9] proposed a system that combines the navigation satellite GPS and the communication constellation Globalstar. This system can quickly solve the integral period ambiguity of the GPS positioning signal. Reid [10] and others proposed an architecture that uses existing LEO broadband satellites to carry payloads and chip atomic clocks to make the functions of these commercial broadband LEO constellations more economic. Joerger et al. [11] integrated iridium constellation and GPS and proposed a residual-based carrier phase receiver autonomous integrity-monitoring algorithm, which can achieve coverage of the continent and navigation integrity. Ke et al. [12] and Ge [13] et al. evaluated and confirmed that the convergence time of Precise Point Positioning (PPP) can be effectively shortened after the integration of GNSS and LEO constellation. Li [14] and others proposed a multi-GNSS algorithm with enhanced full operation capability for LEO constellation navigation to achieve rapid convergence of PPP time. Aiming at the low orbit constellation design for navigation enhancement function, Tian [15] proposed a combined constellation design to achieve global coverage in combination with space garbage distribution, collision risk, coverage, and economy. Han [16] and others designed a global polar orbit constellation and discussed the connectivity and robustness of the inter-satellite link topology of the constellation. Shen [17] proposed a low-orbit satellite navigation enhancement system based on the integration of conduction and guidance, which realized the autonomous precise orbit determination of low-orbit satellites and effectively improved the spatial geometric accuracy factor (GDOP) and PPP accuracy of GNSS.

At present, the research on LEO navigation constellation mainly focuses on the constellation coverage characteristics, positioning accuracy, and convergence time. Although these requirements overlap with the traffic control requirements under the smart city scenario, the urban environment has its particularity. The increasing number of high-rise buildings makes the average elevation angle of urban users higher [18], which has a great impact on the visibility of satellites. In addition, the densely populated area where the city is located needs more coverage and a shorter revisit time. Moreover, due to the low orbit of the satellite and the small ground coverage of a single satellite, how to meet the global and key regional services with fewer satellites also needs to be considered. More importantly, it is difficult to accurately determine and forecast the orbit of low-orbit satellites. Table 1 shows the acceleration magnitude of the orbital perturbation force at different heights. It can be seen that the atmospheric resistance and the Earth’s non-spherical gravitational perturbation to the low orbit are very obvious. Due to the large air resistance in LEO, both the satellite speed and the orbital height will decrease. Frequent startup is required for orbit maintenance, but the amount of fuel carried is limited, hence, shortening the lifespan of the LEO satellite [19].

In related works, although in most cases, atmospheric drag is usually regarded as a disturbance that must be overcome, under specific conditions, atmospheric drag can also be used to control satellite orbit. Dutoit [20] verified the theoretical feasibility of low-orbit constellation configuration control using atmospheric drag. In recent years, atmospheric drag has been widely used in low-orbit satellite flight formation control. According to the problem of formation configuration maintenance using atmospheric drag, Ivanov and Larbi [21,22] studied the relative motion control algorithm based on the linear quadratic regulator and the combined feedforward and feedback PID control law. Lambert et al. [23] verified the feasibility and effectiveness of using atmospheric drag to maintain a low-orbit satellite formation configuration. Traub et al. [24] studied the compensation method for atmospheric perturbation by combining new aerodynamic material with an atmospheric breathing electric propulsion system. Zhao et al. [25] proposed an integrated attitude and orbit control design by changing the attitude to adjust the area-mass ratio for the long-term stability of the accompanying satellite. Kumar et al. [26]. proposed a formation configuration maintenance method using environmental perturbation—namely atmospheric drag and solar light pressure—for low-orbit satellites and synchronous orbit satellites. Sun [27] proposed an attitude orbit-coupling control model with six aerodynamic plates as actuators. Xiaowei et al. [28] proposed a feedback control law based on nonlinear Lyapunov based on five aerodynamic plates. However, due to the different mission objectives of formation flying and constellation maintenance, the requirements for configuration are also different. For constellation configuration maintenance, Lin [29] verified the feasibility of aerodynamic constellation control and studied its optimal control problem. On the basis of using atmospheric drag, Xiang [30] used the maximum and minimum ballistic coefficients of satellites to maintain constellation configurations. Although the above method is theoretically feasible by adjusting the satellite attitude and using the extreme value of the satellite surface-to-mass ratio as the control quantity, the consideration of the constraints of the energy consumption, rotation ability, and three-axis stabilized satellite attitude pointing that is widely used in LEO constellation applications is still insufficient.

This paper is inspired by the requirements and constraints of traffic control issues under a smart city scenario. It surveys the existing literature and defines the explicit needs of navigation services such as navigation accuracy, global coverage, multiple coverages, continuous coverage for key areas, and DOP value. By considering the constraint of user elevation angle in a dense urban area together with the requirements above, the analysis of single satellite coverage and the coverage band of multiple satellites is conducted, and therefore, determines the number and configuration constellation. Moreover, to alleviate service disruption, attitude instability, and energy consumption caused by active station-keeping maneuvers, this paper proposes a control strategy of using atmospheric drag to maintain the relative configuration. Based on the periodic orbit propagation, the angular velocity of the controlled target satellite is adjusted and aligned with the reference satellite by periodically rotating the solar panel to change its effective frontal area, rather than simply applying extreme value. Hence, the atmospheric drag can be used to maintain the relative configuration of the constellation rather than initiating thrusters.

The contributions of this paper are as follows:

- This paper clarifies the requirements of the design and maintenance of the navigation constellation traffic control problem under a smart city scenario and identifies the research gap in the aspect of the LEO navigation constellation design of the existing literature. With joint consideration of the constraint of the elevation angle and coverage in a smart city scenario and atmospheric perturbation, the paper proposes the configuration of an LEO navigation constellation that not only achieves global sensing coverage, but also provides continuous lane-level navigation service with multiple coverages for the key area.
- This paper explores the research gap in constellation station keeping using atmospheric drag of the existing literature. With such consideration of low orbits and the constraint of satellite power and attitude control, a method is further proposed by rotating solar panels to change the effective frontal area of the satellite to achieve relative configuration maintenance of the LEO constellation without initiating active maneuver.
- This paper validates the design of an LEO navigation constellation by checking its navigation accuracy, coverage multiplicity, coverage continuity, and GDOP value, in order to ensure the design meets the needs of navigation services in a smart city scenario. It also validates the control strategy of constellation maintenance by running a one-year simulation. The results prove the efficiency of the proposed strategy, which can provide stable relative phase-keeping with consideration of the constraint of energy absorption and rotation ability of the solar panel, as well as the attitude stability of a single satellite.

The rest of this paper is organized as follows: In Section 2, key elements of constellation design are discussed. In Section 3, the constraints and requirements of navigation in a smart city scenario are defined. In Section 4, the perturbation of Low Earth Orbit is analyzed. In Section 5, the constraint of solar panel rotation ability and energy absorption is discussed. In Section 6, the maintenance strategy of the constellation configuration is presented. In Section 7, the simulation results of constellation coverage and the station-keeping strategy are analyzed. Finally, Section 8 concludes the paper.

## 2. Analysis of Key Elements in Constellation Design

The configuration of a constellation is often described in N/P/F, where N is the total number of satellites, P is the number of orbital planes, and F is the phase factor. The determination of configuration is largely affected by the following key elements.

### 2.1. Orbit Height

The setting of the orbit height depends on many factors. If the altitude is too high, the signal attenuation is serious, which affects signal transmission. If the altitude is too low, the satellite would be hugely affected by atmospheric perturbation and the orbit would suffer a large descent. In addition, special consideration should be given to the influence of the Van Allen radiation belt. Its internally charged particle density is high, its radiation is strong, and it is destructive to electronic components. It is divided into the inner zone (1500 km to 6000 km from the ground) and the outer zone (13,000 km to 20,000 km from the ground). Therefore, the orbital height must avoid the radiation zone. At present, the common low-orbit satellite constellations on the market are all less than 1500 km high: the Iridium satellite is 780 km, OneWeb is 1200 km, and Globalstar is 1450 km. Based on atmospheric perturbation, signal attenuation, coverage, and other factors, 1000 km is selected as the altitude of the LEO enhanced navigation constellation.

### 2.2. User Elevation Angle

**Figure 1:**
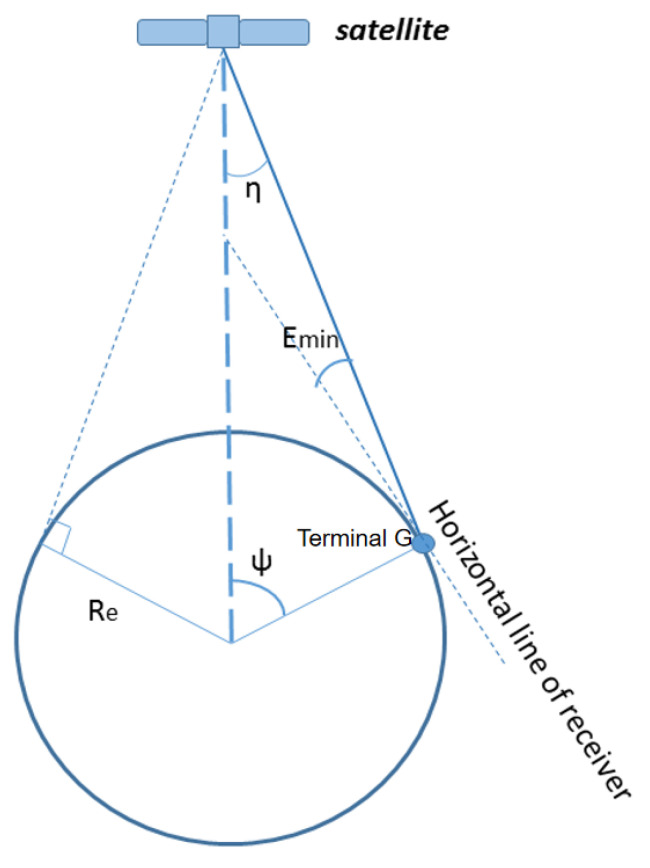
Schematic diagram of satellite coverage angle.

The coverage area of the satellite is greatly related to the orbit height and user elevation angle. For user terminal G, the minimal elevation angle is Emin, the visible satellite is S, and then a triangle is formed by the user, satellite, and geocenter. The relationship between the user’s minimum elevation angle, orbital height, and coverage angle is shown in Figure 1.

According to the trigonometric function, the relation between the user’s minimum elevation, orbital height, and the geocentric angle of the coverage area can be expressed as:

(1) $$\psi =\arccos\frac{R_{e}\cos E_{\min}}{R_{e}+h}-E_{\min},$$

where ψ is the satellite coverage angle, $R_{e}$ is the Earth’s radius, h is the orbital height, and $E_{\min}$ is the minimum user elevation angle. From the formula above, the larger the user minimum elevation angle, the smaller the coverage angle; therefore, more satellites are needed. However, in the smart city scenario, the minimum elevation angle of users is generally large. To ensure coverage continuity, the total number of satellites inevitably increases.

The single satellite visual time t can be expressed as:

(2) $$t=\frac{T\psi }{\pi },$$

where the orbital period T can be expressed as:

(3) $$T=2\pi \sqrt{\frac{a^{3}}{\mu }}.$$

### 2.3. Orbital Inclination

A total of 90% of the world’s population is concentrated in the northern hemisphere, and 50% of the world’s population is concentrated between 20° and 40° north latitude. The LEO navigation constellation should provide better navigation services for densely populated areas while providing global coverage. The vast majority of smart cities in the world are also distributed in this area, and the inclination is determined by the maximum and minimum latitude of the area that needs continuous coverage:

(4) $$i=\varphi_{\min}+\frac{\varphi_{\max}-\varphi_{\min}}{2},$$

where $\varphi_{\min}$ is the minimal latitude of the required coverage area, $\varphi_{\max}$ is the maximum latitude of the required coverage area.

### 2.4. Single Satellite Coverage

The coverage area of the satellite is greatly related to the satellite coverage angle. The coverage area of a single satellite can be expressed as:

(5) $$S=2\pi R_{e}(R_{e}-R_{e}\cos\psi ).$$

When multiple satellites cover seamlessly, each satellite coverage area can be approximated to a spherical hexagon.

(6) $$A{=6R}_{e}^{2}\left(2\arctan\frac{\sqrt{3}}{\cos\psi }-\frac{2\pi }{3}\right).$$

The surface area of the Earth is $A=4\pi R_{e}^{2}$; thus, the total number of satellites with the least global coverage can be expressed as:

(7) $$N=\frac{\pi }{3\arctan\left(\frac{\sqrt{3}}{\cos\psi }\right)-\pi }.$$

### 2.5. Street of Coverage Composed of Multiple Satellites

The Walker constellation design method proposed by J. G. Walker [31] and the SOC (street of coverage) constellation design method proposed by Ullock and Schoen [32] are highly recognized as analytic design methods to achieve large-scale continuous global coverage of circular orbit satellite constellations.

**Figure 2:**
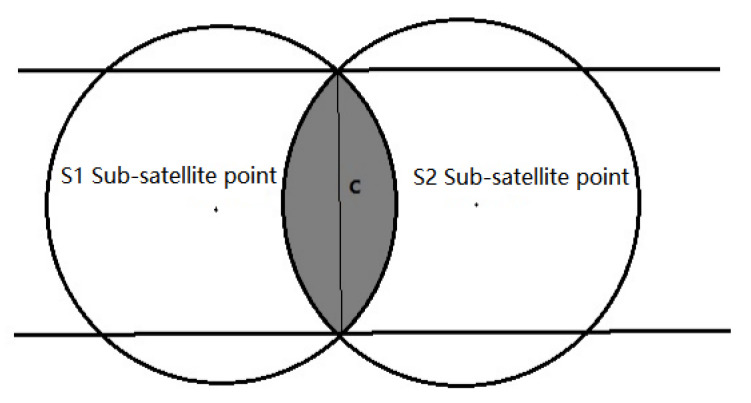
Street of coverage formed by two satellites.

The SOC analysis method can only be used when the satellites in each orbit are evenly distributed in the orbital plane, and the number of satellites in each orbit is not less than three. The coverage areas of each satellite overlap on the ground and form a street. The coverage street formed by the two satellites is shown in the Figure 2.

The width $C_{1}$ of the coverage band formed by S satellites can be expressed as:

(8) $$C_{1}=2\arccos\left[\frac{\cos\psi }{\cos\left(j\pi /s\right)}\right],$$

where j is the number of multiple coverages, and j≤S−1 and S are the total number of satellites in the same orbital plane. For multiple coverage zones composed of multiple satellites, the coverage area is reduced accordingly. For the coverage belt composed of P orbital planes, the width of the coverage belt can be widened or narrowed by adjusting the RAAN between the adjacent orbital planes. Assuming that the satellites on the two orbital planes move in the same direction, the width of the coverage zone is expressed as:

(9) $$C=\left(P-1\right)\left(\psi +\frac{1}{2}C_{1}\right)+C_{1}.$$

**Figure 3:**
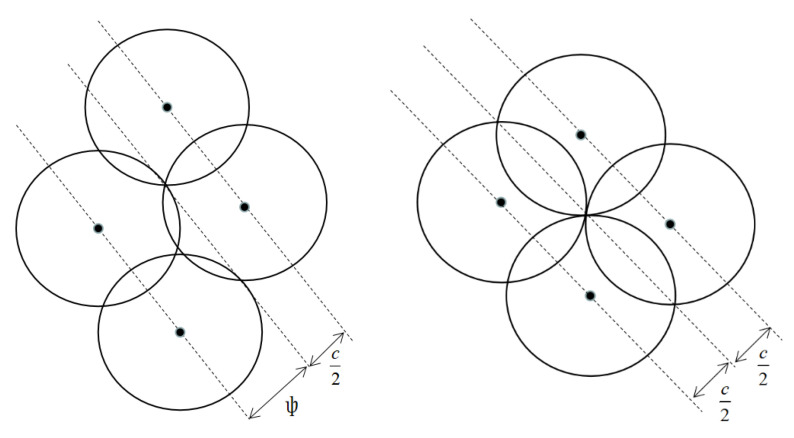
Coverage belt of same direction orbit and reverse orbit.

In the design of SOC, the motion direction of satellites in adjacent orbits is the same, and the motion direction of the first orbital plane and the last orbital plane is opposite, as shown in the Figure 3:

The relation between the same direction orbit and the reverse orbit is as follows:

(10) $$\{\begin{array}{c} \Delta_{same}=\psi +\frac{C}{2}\\ \Delta_{reverse}=C \end{array}.$$

If the constellation is to meet the global coverage, the number of orbital planes on the equator must cover half of the equator.

(11) $$\left(P-1\right)\Delta_{same}+\Delta_{reverse}=\pi .$$

Adding Equation (10) into Equation (11), the relation between the number of orbital planes P, the number of satellites in one orbit S, and the coverage angle ψ of a single satellite is:

(12) $$\left(p-1\right)\psi +\left(p+1\right)\arccos\frac{\cos\psi }{\cos(j\pi /S)}=\pi .$$

## 3. Key Constraints of LEO Navigation Constellation Design in Smart City Scenario

The primary task of the LEO constellation is to ensure global coverage. On this basis, for the need to enhance navigation services, it is necessary to ensure quadruple coverage of the smart city area. In addition, because of the tall buildings in the urban environment, the minimum elevation angle of users is highly constrained, which affects the visibility of satellites in this area. The constraint of the user’s minimum elevation angle will further affect the orbital height, thereby generating an effect on the total number of satellites in the constellation, the number of orbital planes, the coverage angle of a single satellite, and the width of street coverage. In addition, the requirements of LEO navigation for multiple coverages will also affect the width of the coverage band on the above basis. The requirements for continuous services in key areas of smart cities will also affect the inclination and type of constellation.

### 3.1. Navigation Accuracy

The traditional navigation application is road level, represented by TBT (turn-by-turn) navigation that relies on continuous monitoring, which requires a positioning accuracy of about 10 m. A more sophisticated navigation experience, such as lane-level navigation, requires the car to be positioned on the lane, which requires a position accuracy of about 1 m. For intelligent driving scenarios, in order to ensure the safety of automatic driving, the requirements for navigation accuracy are higher. Generally, the accuracy in the transverse direction of the road needs to be less than 1 m. The traffic control under the smart city scenario requires precision not only to reach the lane level, but also to meet the intelligent driving scenario as much as possible. This generates higher requirements for the design of the LEO navigation constellation.

### 3.2. User Elevation Angle

The elevation of satellites at certain specific locations has a critical impact on their applications. For example, for a period of time, satellite ground stations are not able to receive signals from a low-elevation satellite. There are two main reasons: first, compared with signals from high-elevation satellites, signals from low-elevation satellites have a longer path through the dense atmosphere, which makes the signal intensity attenuation more serious; secondly, some objects on the horizon (such as high-rise buildings) may be located between the ground station and the satellite, which blocks the transmission of satellite signals. In a densely built smart city, high-rise buildings block the transmission of satellite navigation signals between the receiver and low-elevation satellites. In the worst case, they can even block communication between satellites with an elevation of 70° and the ground. Therefore, signal receivers and transmitters in cities are generally installed on the top of buildings. However, for vehicles driving on the road, interference on the signal propagation path is inevitable, and the user elevation angle will be relatively large. Therefore, the design of the LEO navigation constellation must give priority to the constraint of the user’s large elevation angle.

Based on the previous SOC analysis method, the total number of satellites, minimal user elevation angle, and orbital height have a greater impact on the constellation design. The determination of the minimum user elevation angle and orbital height has a decisive influence on coverage performance.

The total number of satellites determines the total cost of the constellation, and its parameter design is directly related to the orbital height and the minimum ground elevation. When the number of satellites decreases, it is necessary to increase the orbital height or reduce the minimum user elevation angle to meet the same coverage performance. On the contrary, the increase in the number of satellites will lead to a decrease in the orbital height or an increase in the minimum user elevation angle. On the other hand, the large number of satellites is conducive to improving the visibility of satellites at high elevations and enhancing service performance in the smart city environment. The average user elevation chosen in this article is 45°, and the orbital height is 1000 km. According to Equation (1), the coverage angle is 7.3°.

### 3.3. Global Continuous Coverage

In addition, to ensure global coverage, the minimum number of satellites in the constellation can be calculated as 296 according to Equation (7). Considering satellite redundancy and backup, the total number of satellites shall not be less than 300. For the requirement of global coverage, there must be at least three track planes within 360°. Hence, P≥3; to ensure continuous coverage, the number of satellites in each orbit must also be greater than or equal to 3, and the total number of satellites N=P×S.

### 3.4. Multiple Coverage

Ballard believes that if all satellites in the constellation adopt circular orbits of the same height, at least 2n + 3 satellites will be required to achieve global n-time coverage [33], while at least four-times coverage will be required to achieve LEO navigation missions, with a total of at least 11 satellites. However, the number is far more than that. According to Equation (8), under the premise that the known coverage angle is 7.3°and four-times coverage is guaranteed, S should not be less than 100, that is, each orbital plane should have at least 100 satellites. According to Equation (12), in order to ensure the continuous coverage of the equatorial surface, the orbital planes should not be less than 22. Therefore, the number of orbital planes P is 22, the number of satellites per orbit S is 100, and the total number N reaches 2200.

### 3.5. Continuous Coverage of Key Areas

Since 2000, GaWC (Globalization and World Cities Study Group and Network), a global authoritative city rating agency, has published the 2022 global city ranking by measuring 13 aspects—including international popularity, population base, transportation, cultural institutions, and geographical location—through examining the development of urban finance, professional fields, innovation knowledge flow, etc. The top ten are New York, London, Tokyo, Paris, Singapore, Los Angeles, Hong Kong, Shanghai, Sydney, and Toronto. These cities have already embraced and implemented the concept of smart cities, and all of them are located between 1° N and 51° N. Therefore, the inclination setting must cover this range. To ensure that the latitude range can cover the poles at the same time, 55° is selected as the constellation inclination in this paper.

### 3.6. DOP Analysis

In GNSS, the geometric accuracy factor DOP (Dilution of Precision) is an important indicator to measure the rationality of satellite constellation design and navigation performance. It is used to measure the impact of the spatial geometric distribution of observation satellites on positioning accuracy. The DOP value is in direct proportion to the navigation and positioning error. The larger the DOP value, the greater the positioning error, and the lower the positioning accuracy [34]. DOP is divided into the following categories: *PDOP* (Position Division of Precision), which is the root value of the square sum of errors such as latitude, longitude, and altitude; *TDOP* (Time Division of Precision) clock error precision factor, which is the deviation error value of the time meter in the receiver; *HDOP* (Horizontal Division of Precision) horizontal component precision factor, which is the root value of the square sum of errors such as latitude and longitude; and *VDOP* (Vertical Division of Precision). Their relation can be expressed as:

$$HDOP^{2}+VDOP^{2}=PDOP^{2}$$

$$PDOP^{2}+TDOP^{2}=GDOP^{2}$$

The position accuracy factor *PDOP* (Position Dilution of Precision) directly reflects the distribution of navigation satellites. When the *PDOP* is large, it indicates that the geometric distribution of the four navigation satellites in the air is not ideal. If the perimeter of their figure is too short, the positioning accuracy is low. Generally, the better the satellite distribution, the smaller the *PDOP* value, which is generally less than 3. Geometric Dilution of Precision (*GDOP*) is a very important coefficient to measure positioning accuracy. It represents the range vector amplification factor between the receiver and the space satellite caused by the GPS ranging error. The larger the *GDOP* value is, the worse the navigation accuracy will be. Therefore, in constellation design, especially in the area between 1° N and 51° N, the value of *GDOP* should be as small as possible.

## 4. Perturbation Analysis of Low Orbit

After the deployment of the constellation, to achieve continuous global coverage, it is necessary to maintain the predetermined geometric configuration, that is, to maintain a certain relative position between the satellites in the constellation. Due to the existence of various perturbation forces and initial positioning errors in the real situation, the relative position between satellites constantly shifts. This offset accumulates continuously and finally affects the configuration of the constellation, resulting in the failure of global continuous coverage. Therefore, during the lifespan of the constellation, it is necessary to control the satellite orbit, which is called the station keeping of the constellation.

The LEO satellite is mainly affected by four types of perturbation forces: non-spherical gravity, the gravity of the sun and the moon, the perturbation caused by the solar light pressure, and atmospheric drag. Under the continuous action of the perturbation force, the satellite cannot operate according to the law of two-body motion, and its orbital elements are also changing constantly, thus, causing the drift of the satellite orbit. Therefore, it is necessary to analyze the perturbation of LEO satellites and maintain the satellite orbit according to its orbital change characteristics. Table 1 shows the types of perturbation and relative magnitude. It can be seen that for LEO satellites, the Earth’s non-spherical J2 perturbation and atmospheric drag potential are the main sources of perturbation, and other perturbations are in small quantities. Therefore, this paper mainly analyzes the Earth’s non-spherical and atmospheric drag.

### 4.1. Non-Spherical Perturbation of the Earth

For LEO satellites, the Earth’s gravity and the J2 term of the Earth’s non-spherical perturbation have a greater impact on the satellite. Their influence on the long-term change rate of satellite orbital elements is expressed as follows:

(13) $$\{\begin{array}{c} \dot{a}=0\\ \dot{e}=0\\ \dot{i}=0\\ \dot{\Omega }=-\frac{3J_{2}R_{e}^{2}}{2p^{2}}n\cos i\\ \dot{\lambda }=\frac{3J_{2}R_{e}^{2}}{2p^{2}}n\left[\left(2-\frac{5}{2}\sin^{2}i\right)-\sqrt{1-e^{2}}\cdot \left(1-\frac{3}{2}\sin^{2}i\right)\right] \end{array},$$

where a is semi-major axis, e is the eccentricity, i is the orbital inclination, and Ω is the right ascension of the ascending point, λ is trace angle, $p=a\left(1-e^{2}\right)$, $n=\sqrt{\frac{\mu }{a^{3}}}$, $R_{e}$ is the radius of the Earth, μ is the Earth’s gravitational constant. Therefore, the gravity and non-spherical J2 perturbation have no long-term influence on the semi-major axis, eccentricity, and inclination of the satellite, but have an obvious influence on the right ascension and along-track angle of the ascending intersection point.

### 4.2. Atmospheric Drag Perturbation

When a satellite in Low Earth Orbit moves in the upper atmosphere at high speed for a long time, the accumulation of atmospheric drag leads to orbital attenuation. The atmospheric drag acceleration can be expressed as:

(14) $$a_{drag}=-\frac{1}{2}C_{d}\frac{s}{m}\rho v.$$

$C_{d}$ is the resistance coefficient, ρ is the atmospheric density at the altitude where the spacecraft is located, s is the frontal area of the satellite, m is the mass of the satellite, and v is the velocity of the satellite relative to the atmosphere. By adjusting the pointing of the solar panel, the frontal area s of the satellite can be changed, thus, changing the perturbation of the satellite by atmospheric drag.

## 5. Constraint of Solar Panel Rotation and Energy

**Figure 4:**
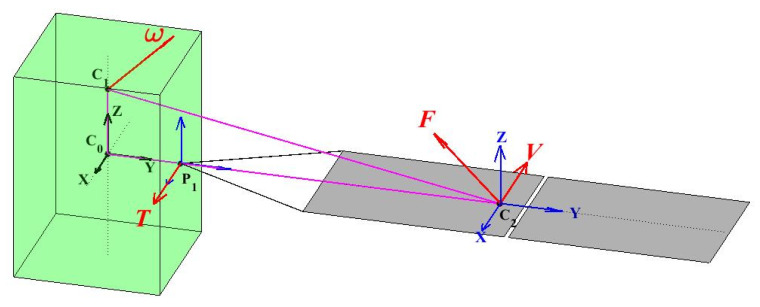
Schematic diagram of solar panel motion and root hinge stress analysis.

Changes in mass characteristics caused by the rotation of the solar panel also need to be considered. Therefore, it is necessary to analyze the force acting on the rotational motion of the solar panel and its root hinge. The schematic diagram of the motion of the solar panel and the force analysis of its SADA root hinge is shown in the Figure 4. 

Where $C_{0}$ is the initial centroid position of the satellite; $C_{1}$ is the actual position of the satellite after the centroid offset at a certain time; $C_{2}$ is the centroid position of the solar panel (including the connecting frame); $P_{1}$ is the position of the SADA installation point; ω is the angular velocity vector of the satellite at a certain time; V is the velocity vector of the solar wing at the center of mass; F is the centripetal force vector of the solar wing at the center of mass; and T is the torque vector of the SADA root hinge under the action of the centripetal force vector of the solar panel.

The whole satellite reference coordinate system $C_{0}-XYZ$ is established with $C_{0}$ as the origin; the SADA installation coordinate system $P_{1}-XYZ$ is established with $P_{1}$ as the origin; and the reference coordinate system $C_{2}-XYZ$ of the solar panel centroid is established with $C_{2}$ as the origin. The SADA axis of the solar panel is always consistent with its *y* axis, so that $m_{1}$ is the mass of the unilateral solar panel (including the connecting frame).

In 3D space, the following operations can be performed according to the knowledge of classical mechanics and vector calculation:

(15) $$V=\omega \times \vec{C_{1}C_{2}}=\omega \times \left(\vec{C_{1}C_{0}}+\vec{C_{0}P_{1}}+\vec{P_{1}C_{2}}\right),$$

(16) $$a=\omega \times V=\omega \times \left(\omega \times \left(\vec{C_{1}C_{0}}+\vec{C_{0}P_{1}}+\vec{P_{1}C_{2}}\right)\right),$$

(17) $$T=F\times \vec{C_{2}P_{1}}=m\cdot a\times \vec{C_{2}P_{1}}=m_{1}\cdot \omega \times \left(\omega \times \left(\vec{C_{1}C_{0}}+\vec{C_{0}P_{1}}+\vec{P_{1}C_{2}}\right)\right)\times \vec{C_{2}P_{1}}.$$

During the satellite operation, the energy required by the satellite depends on the solar panels. Since the area of the solar panel is constant, the amount of energy it can provide directly depends on the angle between the normal vector of the solar panel and the solar vector [35].

**Figure 5:**
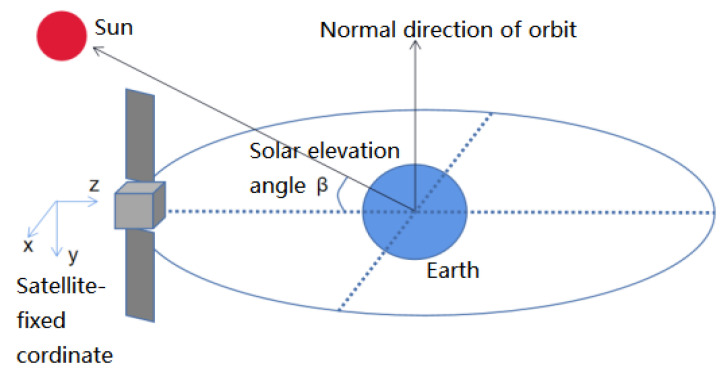
Schematic diagram of yaw maneuver of three-axis stabilized satellite.

The three-axis stabilized satellite adopts dynamic yaw control in order to keep the antenna surface pointing to the ground and the sun directly shining on the solar panel. For the three-axis stabilized satellite, its attitude stability is controlled by the dynamic yaw control mode. Figure 5 describes how the three-axis stabilized satellite conducts yaw control. 

With the center of the mass orbital coordinate system as the benchmark, the Euler angle is used for positioning, that is, the *x* axis, *y* axis, and *z* axis. The *x* axis is the rolling axis, the *y* axis is the pitching axis, and the *z* axis is the yaw axis. When the solar altitude angle β is large, although the solar panel can rotate at a specific direction to track the sun and can meet the accuracy requirements, it cannot meet the energy supply requirements of the whole satellite.

Therefore, based on actual engineering practice, in order to ensure the sufficient energy supply for the satellite, a certain amount of energy margin is reserved in the satellite design. Thus, under the overall energy demand, the rotation of the solar panel within a certain angle will not affect the normal operation of the satellite. The relationship between the actual energy absorption e and the normal direction of the sail and the solar vector α can be expressed as:

(18) e=E×cosα. 

E is the energy absorption when the solar panel is fully oriented to the solar vector. Under normal engineering constraint, $\alpha =\pm 5^{\circ }$. However, this is because the solar panel also needs to achieve the function of using atmospheric drag to maintain phase at the same time. Hence, a relation between energy consumption and α is designed in Table 2.

Therefore, when designing the control rate of the rotation of solar panel, it is necessary to consider the energy consumption. According to the engineering experience, the energy consumption of 30% is taken as the maximum constraint of the angle of the solar panel.

## 6. LEO Constellation Configuration Maintenance

For satellites that share the same orbital height, eccentricity, and inclination, the changes in the RAAN caused by the Earth’s oblateness perturbation are the same. For constellations composed of satellites of the same orbital type, the long-term drift of the RAAN caused by the changes in the RAAN will cause the constellation to drift as a whole, and for regional constellations, this will cause the systematic drift away from their service areas. However, it will not affect the coverage and services of the global constellation. According to the research of Yun et al. [36] on the control frequency of large constellations at different altitudes, the constellations with low altitudes are more suitable for using the relative configuration maintenance method. Therefore, this paper follows the above idea and maintains the relative position relationship between all satellites by adjusting the semi-major axis of the orbit. With the absolute longitude of the satellite drifts, the overall configuration of the constellation can remain stable. Compared with the absolute configuration maintenance method, the control efficiency of the relative configuration maintenance method is higher; the requirements for launching the satellite into orbit are relatively low; and the global coverage of the constellation is basically fixed, which is conducive to the constellation performance analysis.

### Station Keeping Strategy

First, the mathematical relation between the semi-major axis and the satellite frontal area is established. The satellite angular velocity is expressed by the semi-major axis of the orbit:

(19) $$n=\sqrt{\frac{\mu }{a^{3}}}.$$

The angular acceleration of the satellite is:

(20) $$\dot{n}=-\frac{3}{2}n\frac{\dot{a}}{a}.$$

The relationship between semi-major axis variation and the satellite frontal area is:

(21) $$\dot{a}=-C_{d}\frac{s}{m}na^{2}\rho ,$$

where $C_{d}$ is the resistance coefficient, ρ is the atmospheric density at the altitude where the spacecraft is located, s is the frontal area of the satellite, and m is the mass of the satellite.

According to Equations (18) and (19), the angular acceleration of the satellite can be expressed as:

(22) $$\dot{n}=\frac{3}{2}C_{d}\frac{s}{m}\frac{\mu \rho }{a^{2}}.$$

The angular velocity difference between the two satellites after time t can be expressed as:

(23) $$\Delta u=\Delta u_{1}+\frac{1}{2}({\dot{n}}_{1}-{\dot{n}}_{2})t^{2},$$

where $\Delta u_{1}$ is the initial phase difference between two satellites.

Substituting Equation (22) into Equation (23), it can be found that the key variable determining the phase difference is the angular velocity difference; the relation is:

(24) $$\Delta u=\Delta u_{1}+\frac{1}{2}\Delta nt.\;$$

Therefore, Δn can be used as the main variable to control the phase difference, and then the relationship between the frontal area and Δn can be established. Substituting Equation (21) into Equation (22), the relationship between the frontal area of the S2 satellite and the speed difference between the two satellites can be obtained:

(25) $$S_{2}=\frac{a_{2}^{2}(3tC_{d}\mu \rho s_{1}-2\Delta nma_{1}^{2})}{3TC_{d}\mu \rho a_{1}^{2}},\;$$

where $S_{body}\leq S_{2}\leq S_{body}+S_{panel}$.

**Figure 6:**
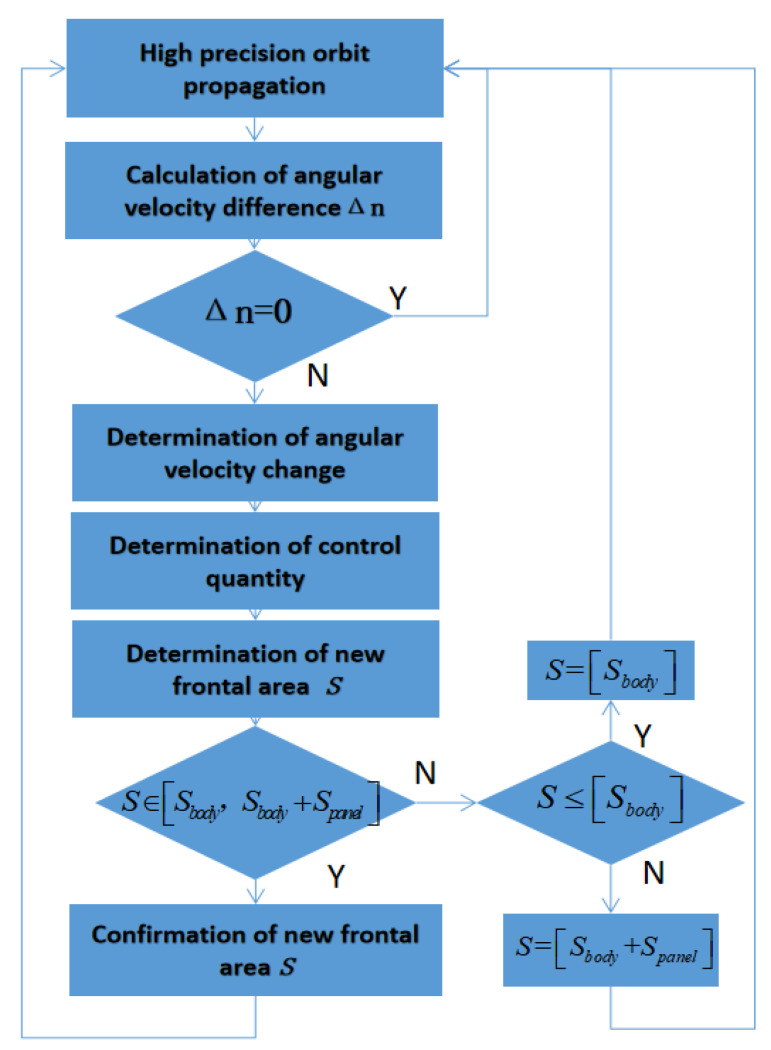
Flow chart of station-keeping control strategy.

On the basis of the above mathematical relationship, the flow of the control strategy of the solar panel is shown in the Figure 6.

As an example, take the two satellites with different phases and different frontal areas at the initial time but located in the same orbital plane.

(1) High-Precision Orbit Propagation

After obtaining the real-time orbit data of two satellites, HPOP (High-Precision Orbit Propagation) is carried out, and the propagation period is one orbital period.

(2) Calculation of angular velocity difference

Calculate the angular velocity difference between two satellites in the next period.

The angular velocity difference between the two satellites after T time is:

$$\Delta n=\left({\dot{n}}_{1}-{\dot{n}}_{2}\right)T$$

(3) Determination of conducting phase control

When the angular velocity difference between two satellites is 0, the S2 satellite is not controlled. When the angular velocity difference between the two satellites is not 0, the angular velocity control is achieved.

(4) Determination of angular velocity change

First, calculate the phase difference between the two satellites as the input. It can be seen from Equation (21) that there is a positive correlation between the frontal area and the angular acceleration. If the angular velocity of satellite S1 is greater than that of satellite S2, the frontal area of S2 needs to be increased. If the angular velocity of S1 is less than that of S2, the frontal area of S2 needs to be reduced.

(5) Determination of control quantity

The method is expected to reduce the rotation range of the solar panel as much as possible. Due to the restriction of the effective frontal area, it is necessary to determine the control time range. The phase control adopts high frequency with a small amount of control; this control is achieved according to the frequency time propagated from the orbit, which is the satellite orbital period. The new frontal area required to correct the phase difference for the next period is obtained by substituting into Equation (20).

(6) Determination of new frontal area

If $S^{\prime }\leq \left[S_{body}\right]$, the effective frontal area of the satellite in this orbit is equal to the satellite body area. If $S^{\prime }\geq \left[S_{body}+S_{panel}\right]$, the effective frontal area of the satellite in this orbit is equal to the satellite body plus the maximum solar panel area. When $S^{\prime }\in \left[S_{body},\;S_{body}+S_{panel}\right]$, confirm the new frontal area. Otherwise, $S^{\prime }$ will remain still until the recalculation based on the next orbital propagation.

## 7. Result Analysis and Discussion

According to the previous analysis, the configuration of the constellation in this paper is 2200/22/1, with an orbital height of 1000 km, an orbital inclination of 55°, and a simulation environment of two days. First, the global coverage performance of the constellation and the global GDOP value distribution is simulated and analyzed. To show the advancement of the proposed design, we also apply the Iridium constellation mentioned in the introduction with the same constraints and run the simulation in same manner.

**Figure 7:**
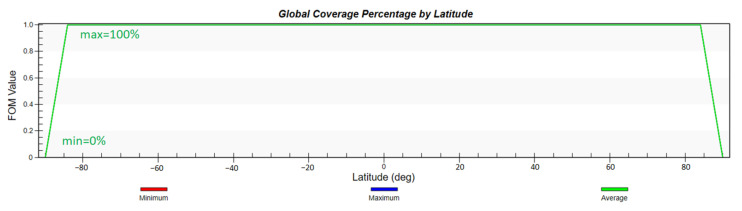
Global coverage percentage by latitude.

**Figure 8:**
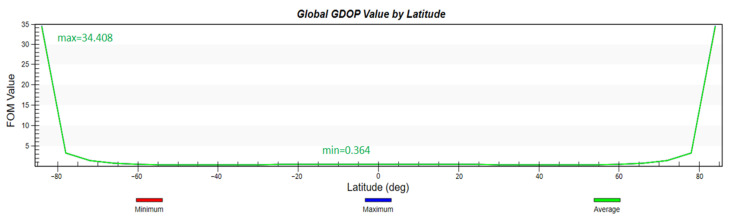
Global GDOP by latitude.

It can be seen from Figure 7 and Figure 8 that the constellation can not only basically achieve global coverage, but also achieve 100% coverage for the range from 1° N to 51° N where the smart city is located. The curve of global GDOP is desirable and its minimum value is 0.364. The GDOP value in the key area is also lower than 1.

**Figure 9:**
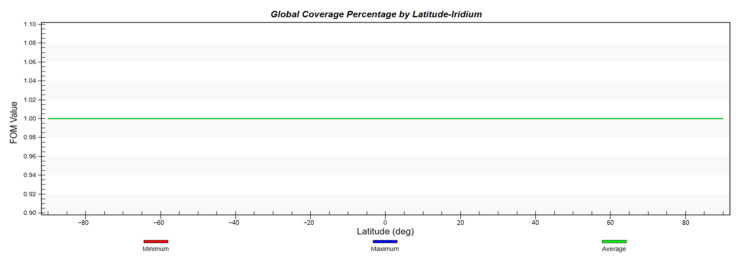
Global coverage percentage by latitude of Iridium constellation.

**Figure 10:**
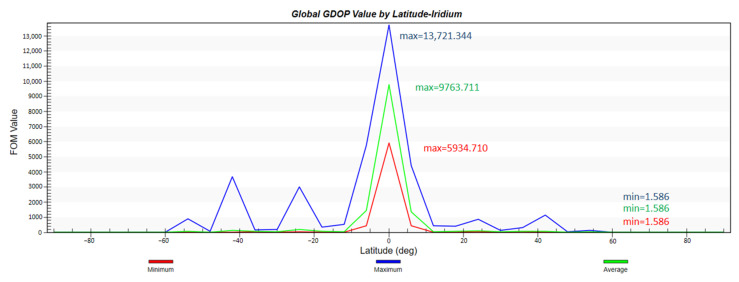
Global GDOP by latitude of Iridium constellation.

Moreover, Figure 9 and Figure 10 indicate that with the help of its larger inclination, Iridium could achieve better coverage in polar areas. However, its GDOP distribution is not ideal, nor is its GDOP average value. The highest average GDOP value appears in the vicinity of the equator and reaches 9763.711. The lowest average GDOP value is 1.568.

Second, in order to evaluate the performance of the LEO navigation constellation in the case of a smart city scenario, the following three dimensions, GDOP value, continuous coverage, and navigation accuracy, are applied to assess five representative smart cities: London, New York, Shanghai, Singapore, and Sydney. The Iridium constellation also runs the same simulation to provide comparative results.

It can be seen from Table 3 that this constellation can not only cover the above cities without interruption, but also, their GDOP values are far less than 1. In addition, the navigation accuracy is far lower than the road-level standard of 10 m, which is close to the lane-level navigation accuracy requirements. However, in Table 4, the coverage performance of the Iridium constellation configuration is far below the established requirements, which is not acceptable for users in a smart city.

Finally, taking London, which has the highest latitude in the Northern Hemisphere, and Sydney, which has the highest latitude in the Southern Hemisphere, as examples, the coverage multiplicity of the two places is simulated. The Iridium constellation also runs the same simulation to provide comparative results.

**Figure 11:**
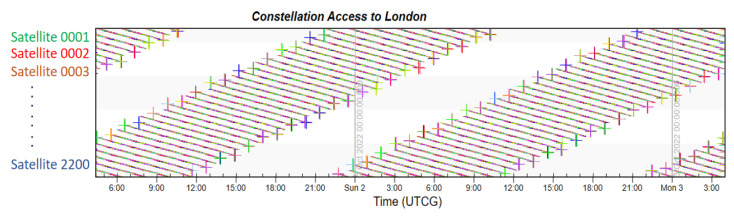
Constellation access to London.

**Figure 12:**
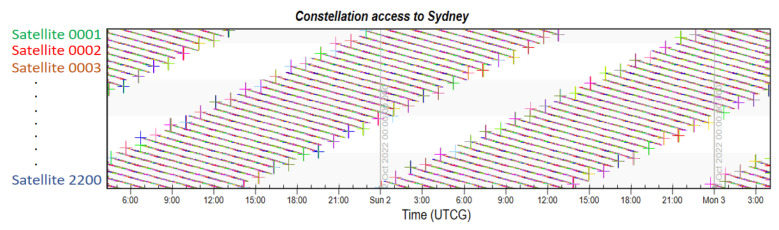
Constellation access to Sydney.

From Figure 11 and Figure 12 showing the constellation access of both cities, each colored dot displayed in accordance with time represents the access availability. There is no doubt that the LEO navigation constellation could provide more than four-times coverage at any second. The simulation indicates that every moment, the average number of visible satellites that fly by London is 175.1, and that number in Sydney is 183.5. This large number not also ensures navigation accuracy, but also guarantees the redundancy of local services.

**Figure 13:**
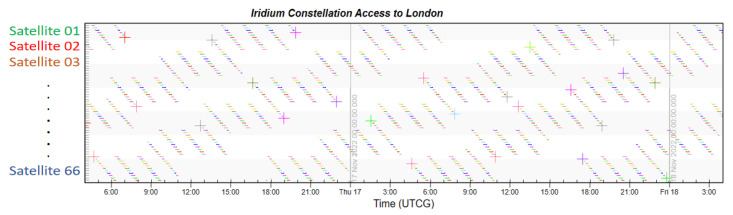
Iridium constellation access to London.

**Figure 14:**
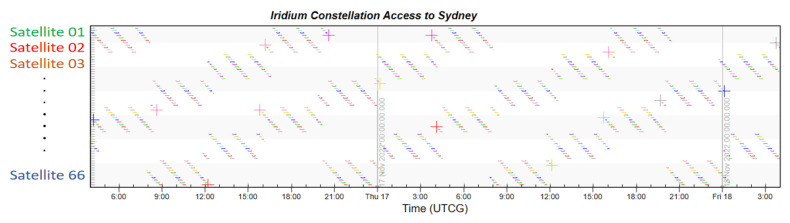
Iridium constellation access to Sydney.

From Figure 13 and Figure 14, we can see that access to both cities is sparsely distributed, which is the result of fewer satellites. The simulation shows that the average number of visible satellites in London is 4.1, which just meets the four-time coverage requirement for navigation service. The average number of visible satellites in Sydney is 2.9, which cannot fulfill the basic demand of the navigation system. The small amount of satellites greatly restrains the coverage performance of Iridium, and it cannot compete with the proposed constellation design.

In terms of constellation configuration maintenance strategy, the period of simulation of the phase keeping of two satellites in the same orbital plane is one year. The following Table 5 shows the initial parameters of two satellites.

**Figure 15:**
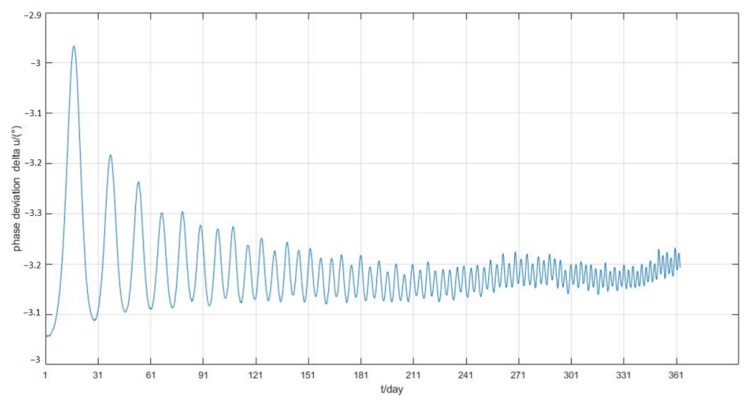
Phase deviation of two satellites.

**Figure 16:**
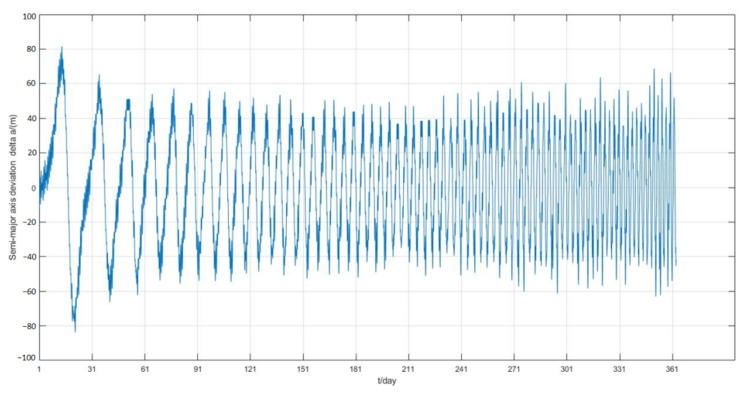
Semi-major axis deviation of two satellites.

**Figure 17:**
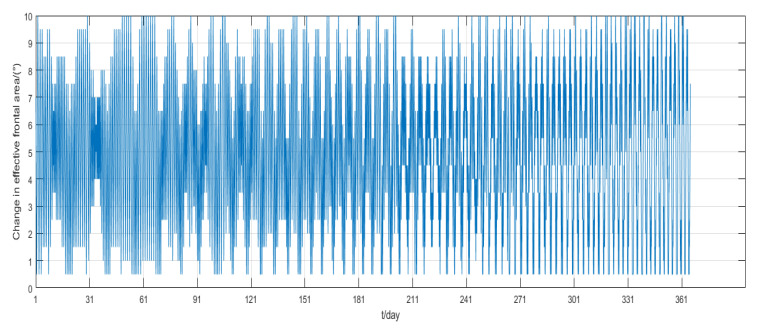
One-year change of frontal area.

The simulation process mainly considers the non-spherical gravitational perturbation and the atmospheric drag perturbation. The simulation results are shown in Figure 15, Figure 16 and Figure 17.

The one-year simulation shows that stable phase keeping can be achieved by adjusting the frontal area of the panel from the Figure 15, and the phase difference between the two satellites is always less than 0.6°, which is far less than the maximum permissible deviation. After 150 days, the phase deviation oscillates within 0.1°. Moreover, from Figure 16, the semi-major axis deviation is always within 80 m, and the value converges to 60 m after nearly 40 days. Figure 17 indicates that the rotation of the solar panel is determined by the change of angular velocity, rather than simply the use minimum or maximum value. The control frequency of the solar panel is determined by the period of orbit propagation, so that it can reach the above control accuracy.

## 8. Conclusions and Future Work

This article analyzes the constraints and requirements of traffic control in a smart city scenario. A LEO navigation constellation is designed; the simulation indicates that its navigation accuracy reaches lane-level accuracy, and the satellite number reaches 2200 due to the serious limitations of the minimal user elevation angle. By comparing with the existing LEO constellation, Iridium, the superiority of the proposed design is proved. The constellation can not only fulfill global coverage with a low GDOP value, but also provide more than four-times coverage multiplicity with almost lane-level navigation accuracy for the key area at every second. The results above are significant for ground vehicles in the smart city scenario.

Moreover, with the help of the relative constellation configuration maintenance concept, a method of the autonomous maintenance of LEO constellation configuration using atmospheric drag is proposed. Based on full consideration of the rotation influence of the solar panel on the satellite mass distribution, the energy impact, and the attitude stability, the satellite station-keeping strategy is implemented effectively on the two satellites from the same orbital plane. During a one-year simulation, the phase deviation of two satellites is less than 0.6°; then, the phase deviation gradually converges to 0.1°. The semi-major axis deviation is less than 80 m during the simulation period. Therefore, by implementing the proposed method, there is no need to initiate active station-keeping control, which could not only decrease fuel consumption, but also increase the continuity of the navigation service to key areas and increase the overall robustness.

## Figures and Tables

**Table 1:** Magnitude of perturbed acceleration of LEO satellite.

Perturbation	Magnitude
Earth gravity	10^1^
Second order non-spherical gravity	10^−2^
Atmospheric drag perturbation	10^−5^
Fourth order non-spherical gravity	10^−5^
Sixth order non-spherical gravity	10^−6^
Lunar gravity	10^−6^
Solar gravity	10^−6^
Solid tidal perturbation	10^−6^
Solar pressure perturbation	10^−7^

**Table 2:** Relation between energy consumption and α angle.

α	Energy Consumption
18.19°	%5
25.84°	%10
36.87°	%20
45.57°	%30

**Table 3:** Table of constellation coverage performance of five smart cities.

City	GDOP	Revisit Time (s)	Navigation Accuracy (m)
London	0.363	0	1.815
New York	0.383	0	1.914
Shanghai	0.404	0	2.018
Singapore	0.469	0	2.342
Sydney	0.399	0	1.994

**Table 4:** Table of Iridium constellation coverage performance of five smart cities.

City	GDOP	Revisit Time (s)	Navigation Accuracy (m)
London	31.592	0	92.802
New York	36.327	0	181.636
Shanghai	20.684	0	103.419
Singapore	2389.914	1788	2243.564
Sydney	70.546	0	164.604

**Table 5:** Initial orbit elements of two satellites.

Simulation Parameters	Value
Mass of satellite	20 kg
Semi-major axis	7378 km
Inclination	55°
Air drag coefficient	2.2
Minimum frontal area	0.5 m^2^
Maximum frontal area	10 m^2^
S1 true anomaly	0°
S2 true anomaly	3.6°
Maximum permissible deviation	3°

## Data Availability

The data presented in this study are available on request from the corresponding author.
